# Population Structure of Endomicrobia in Single Host Cells of Termite Gut Flagellates (*Trichonympha* spp.)

**DOI:** 10.1264/jsme2.ME14169

**Published:** 2015-02-26

**Authors:** Hao Zheng, Carsten Dietrich, Claire L. Thompson, Katja Meuser, Andreas Brune

**Affiliations:** 1 Department of Biogeochemistry, Max Planck Institute for Terrestrial MicrobiologyKarl-von-Frisch-Str. 10, 35043 MarburgGermany; 2 LOEWE Center for Synthetic Microbiology, SYNMIKRO, Philipps-Universität Marburg35043 MarburgGermany

**Keywords:** termites, protists, endosymbionts, cospeciation, pyrosequencing

## Abstract

The gut microbiota of many phylogenetically lower termites is dominated by the cellulolytic flagellates of the genus *Trichonympha*, which are consistently associated with bacterial symbionts. In the case of Endomicrobia, an unusual lineage of endosymbionts of the *Elusimicrobia* phylum that is also present in other gut flagellates, previous studies have documented strict host specificity, leading to the cospeciation of “*Candidatus* Endomicrobium trichonymphae” with their respective flagellate hosts. However, it currently remains unclear whether one *Trichonympha* species is capable of harboring more than one Endomicrobia phylotype. In the present study, we selected single *Trichonympha* cells from the guts of *Zootermopsis nevadensis* and *Reticulitermes santonensis* and characterized their Endomicrobia populations based on internal transcribed spacer (ITS) region sequences. We found that each host cell harbored a homogeneous population of symbionts that were specific to their respective host species, but phylogenetically distinct between each host lineage, corroborating cospeciation being caused by vertical inheritance. The experimental design of the present study also allowed for the identification of an unexpectedly large amount of tag-switching between samples, which indicated that any high-resolution analysis of microbial community structures using the pyrosequencing technique has to be interpreted with great caution.

The termite hindgut is colonized by dense assemblages of prokaryotes (bacteria and archaea) and eukaryotic protists that play essential roles in both digestion and host nutrition (4, 20). The gut microbiota of phylogenetically lower termites is dominated by cellulolytic flagellates that are unique to termites and comprise diverse lineages from the phyla *Parabasalia* and *Preaxostyla* (order *Oxymonadida*) (6). In most cases, these flagellates are associated with large numbers of prokaryotic symbionts that colonize both the surface and cytoplasm (19, 38) and sometimes even the nucleus of their hosts (40). There is strong evidence to show that the symbionts complement the nitrogen metabolism of the flagellates and play an important nutritional role in the hindgut microecosystem (3, 6, 19, 20).

The predominant flagellates in the hindgut of several termite lineages are the hypermastigid flagellates of the genus *Trichonympha*. Originally discovered in *Reticulitermes* spp. (Rhinotermitidae) (16, 29, 30), members of the genus have also been described in several other termite families (including Termopsidae and Kalotermitidae) and in their sister group, the wood-feeding cockroaches of the genus *Cryptocercus* (for references, see 51). While some termite species contain only a single species of *Trichonympha*, others harbor several representatives of this genus.

Termites of the genus *Zootermopsis* harbor at least four *Trichonympha* species. *Trichonympha campanula* (27), *Trichonympha sphaerica* (26), and *Trichonympha collaris* (25) have long been recognized based on differences in morphology; the fourth species was discovered using molecular approaches. The first evidence of the existence of this fourth species was the presence of an additional phylotype of small subunit (SSU) rRNA gene sequences in a cell suspension of *T. campanula* from *Zootermopsis nevadensis* that was differentiated from the cells of *T. campanula* by fluorescence *in situ* hybridization (21). A few years later, this species, *Trichonympha postcylindrica*, was described based on specimens from the gut of *Zootermopsis angusticollis* (45).

Although the same four *Trichonympha* species appear to be present in both *Z. nevadensis* and *Z. angusticollis* (21, 25, 45), their relative abundance may differ even among individuals from the same colony (25). Moreover, there is a considerable range of sequence divergence within the populations of each *Trichonympha* species in *Z. angusticollis* (45). Current knowledge on the diversity of *Trichonympha* species in the two *Zootermopsis* species, including the hitherto unpublished sequence of *T. postcylindrica* from *Z. nevadensis* (21), is summarized in Fig. 1.

Members of the genus *Trichonympha* fall into three distinct phylogenetic clusters (9, 23, 37), and all are associated with bacterial symbionts, quite often more than one bacterial species per flagellate host (39, 40, 44). All members of *Trichonympha* Cluster I harbor large populations of the specific intracellular symbiont, “*Candidatus* Endomicrobium trichonymphae” (22, 36, 43), which belongs to a distinct lineage of uncultured, insect-associated bacteria (“Endomicrobia”) in the *Elusimicrobia* phylum (5). The presence of homogeneous bacterial symbiont populations in a single flagellate host cell (18) and an almost perfect congruence of the phylogenies of the bacterial symbionts and their host flagellates (23) suggest that “*Ca.* Endomicrobium trichonymphae” is propagated exclusively by vertical transmission (cytoplasmic inheritance) in the *Trichonympha* lineage. In this case, the same number of Endomicrobia species as *Trichonympha* species is expected in a given termite. This is in apparent contradiction with the composition of a metagenomic library constructed from DNA prepared from the enrichment of “*Ca.* Endomicrobium trichonymphae” in the gut of *Z. nevadensis*, which contains a larger number of 16S rRNA phylotypes of Endomicrobia than host species (IMG Project ID: Gi01566) (21). It currently remains unclear whether all individuals of each host species harbor the same symbionts and whether a single *Trichonympha* cell carries more than one Endomicrobia phylotype.

To clarify this, we isolated single *Trichonympha* cells from the guts of *Z. nevadensis* and *R. santonensis* and characterized their Endomicrobia populations by high-throughput sequencing, using the internal transcribed spacer (ITS) region between their rRNA genes to resolve even low levels of strain variation within both symbionts and hosts.

## Materials and Methods

### Termites

*Zootermopsis nevadensis* was collected near the Chilao Flats Campground, Angeles National Forest, California, USA in 2006. *Reticulitermes santonensis*, which is synonymous with *Reticulitermes flavipes* (1), was collected near La Gautrelle, Ile d’Oléron, France in 2010. Since then, both species have been maintained in the laboratory on a diet of pine wood and water. Worker termites (pseudergates) were used in all experiments.

### Micromanipulation and whole-genome amplification

Termites were dissected and the entire hindgut content was carefully diluted in Solution U (48) in Eppendorf tubes. Aliquots of this suspension (10 μL) were transferred to the wells of a Teflon-coated microscope slide and inspected with an inverted microscope with phase-contrast optics (50-fold magnification). Individual *Trichonympha* cells were identified by their morphology and captured using a micropipette attached to a micromanipulator, as described previously (47), and transferred to a PCR tube with 50 μL Solution U. The tubes were incubated at 95°C for 10 min to lyse the flagellates, cooled on ice for 2 min, and centrifuged at a slow speed (50 × *g*) for 1 min to remove cell debris. The supernatant (including the endosymbionts) was used as a template for multiple displacement amplification (MDA) with the REPLI-g UltraFast Mini Kit (Qiagen, Hilden, Germany) following the manufacturer’s instructions, except that the reaction time was increased to 4 h.

### Library preparation and sequencing

The ITS regions of flagellates and Endomicrobia were amplified by PCR using the MDA products (25-fold diluted) as a template and specific primer pairs for the proximal regions of the flanking rRNA genes (Fig. 2). The combination of the *Trichonympha-*specific forward primer 18S-Tri-1287f (AAGATTCACGTAGC TGGG; this study) and the universal reverse primer 28S-1r (ATGCTTAAATTCAGCGGGT) (35) yielded PCR products of ~700 bp (30 cycles of amplification: 30 s at 94°C, 30 s at 54°C, and 60 s at 72°C), which were directly sequenced as previously described (47).

The Endomicrobia-specific primers 16S-Endo-1502f (AAGGT AGCCGTACGAGA) and 23S-Endo-28r (ACAGTCTTAGCCAA GGCA) were designed on the basis of all Endomicrobia sequences represented in public databases. Both primers were barcoded as described previously (28). Endomicrobia sequences were also amplified directly from undiluted DNA extracted from the whole-gut homogenates of *Z. nevadensis* and *R. santonensis* (35 cycles of amplification: 30 s at 94°C, 30 s at 56°C, and 60 s at 72°C). All samples were commercially sequenced in a single sequencing run (454 GS FLX 64 with Titanium technology; GATC Biotech, Konstanz, Germany).

### Bioinformatics

The ITS sequences of the selected flagellates were aligned *de novo* with *MAFFT* version 7 (24). After manual curation of the alignment, a neighbor-joining tree was constructed using the *ARB* software suite (31). The ITS sequences of Endomicrobia were processed as previously described (12). Briefly, pyrotag reads with a minimum length of 250 bp and a maximum expected error of 0.5 were selected and demultiplexed using their barcode sequences (no mismatch allowed). After the removal of barcodes and primer sequences, the sequences were clustered at the 99% similarity level with *UPARSE* (13). Sequences were dereplicated, and representative phylotypes (most abundant sequence in the respective cluster) were aligned *de novo* using the *MAFFT* aligner in the L-INS-I mode with 100 iterations (24). Where necessary, the alignment was manually refined so that all sequences were unambiguously aligned. Maximum-likelihood trees were constructed using *RAxML* version 8.1.3 (42) with the 16-state GTR-Γ model and 1,000 bootstraps. A heatmap was generated using the *R* software with the package *heatmap.plus* (11).

### Sequence accession numbers

The ITS region sequences of flagellates have been deposited in GenBank under accession numbers KJ778566–KJ778610. Representative sequences of all Endomicrobia phylotypes obtained from flagellate samples and whole-gut homogenates have been deposited in GenBank under accession numbers KP058245–KP058309.

## Results

### Isolation and identification of single *Trichonympha* cells

We successfully amplified the ITS regions of host flagellates (Fig. 2A) and Endomicrobia symbionts (Fig. 2B) from the whole-genome amplification products obtained from the selected *Trichonympha* cells from *Z. nevadensis* (34 out of 60 cells) and *R. santonensis* (9 out of 20 cells). Direct sequencing of the PCR products obtained with flagellate-specific primers yielded clean signals for all species, except for *T. campanula*, in which multiple bases at several positions of the trace file indicated sequence polymorphism in the ITS region (Supplementary Fig. S1).

A comparative sequence analysis showed that each of the sequences obtained from the morphotypes of *T. sphaerica*, *T. postcylindrica*, *T. collaris*, and *T. campanula* in *Z. nevadensis* clustered with their respective relatives from *Z. angusticollis* (Fig. 3), confirming the morphological assignment of the selected flagellates. The nine sequences from the morphotype of *T. agilis* formed a sister group of the *Trichonympha* species from *Z. nevadensis*.

### Endomicrobia populations in single *Trichonympha* cells

A pyrosequencing analysis of the PCR products obtained with Endomicrobia-specific primers yielded variable read numbers per flagellate sample (after quality control, see Supplementary Fig. S2). Although the number of Endomicrobia phylotypes (99% sequence identity) obtained from all samples (17 phylotypes from 45 single host cells) was markedly larger than the number of flagellate species investigated (5 species), they all clustered more or less closely according to their respective hosts (Fig. 4A).

While the Endomicrobia obtained from nine different cells of *T. agilis* comprised only two closely related phylotypes, the symbionts of the other flagellates, particularly *T. postcylindrica* and *T. campanula*, were far more diverse (Fig. 4A). Phylogenetically distinct flagellates carried different phylotypes of symbionts, and even the Endomicrobia in host cells with identical ITS sequences sometimes differed slightly in their respective phylotype (*e.g.*, *T. sphaerica* ZnvTrn10 and ZnvTrn48).

When we analyzed the diversity of Endomicrobia sequences obtained from the whole-gut DNA of *Z. nevadensis*, we found several additional phylotypes (represented by black branches in Fig. 4A) within the radiation of the Endomicrobia sequences retrieved from selected *Trichonympha* cells, which indicated that the *Trichonympha* populations in this termite were not exhaustively sampled. Nevertheless, the vast majority of the Endomicrobia in *Z. nevadensis* consisted of phylotypes retrieved from *T. collaris* (41%) and *T. sphaerica* (33%), while the diverse populations of Endomicrobia associated with *T. postcylindrica* (13%) and *T. campanula* (6%) represented only a small part of the community (Fig. 4B). In the case of *R. santonensis*, the two phylotypes of Endomicrobia from *T. agilis* accounted for 23% of the phylotypes in the gut homogenate, and the majority of the reads clustered with the ITS sequences of the selected *Pyrsonympha* flagellates (unpublished results), which form a distinct branch in the phylogeny of Endomicrobia (43).

### Pyrosequencing artifacts

Although each of the pyrotag libraries of Endomicrobia from single flagellates consistently contained only one major phylotype (Fig. 5), they always comprised a smaller fraction of reads that were identical with the dominant phylotypes in other libraries. The shared presence of the same Endomicrobia phylotype in different flagellate samples of *Z. nevadensis* may have been caused by the interspecific transfer of endosymbionts within the gut, whereas the presence of identical phylotypes in *Trichonympha* species of different termites was unexpected because it can only be explained by multiple recent transfers of endosymbionts between flagellates of different termite species. Therefore, we suspected that the shared phylotypes were artifacts that either resulted from the picking process (*e.g.*, the symbionts stemmed from other, lysed flagellate cells present in the same gut) or were generated during the pyrosequencing process.

To clarify this, we reanalyzed three of the Endomicrobia samples obtained from individual flagellates (R05, Z13, and Z21), which were selected because they had particularly large proportions of suspicious sequences (see Fig. 5). To exclude the possibility that the minor phylotypes in the corresponding pyrotag libraries stemmed from contamination during the picking process or originated during whole-genome amplification or library preparation (*e.g.*, by contaminated tags), we used aliquots of the samples that had been directly preserved before pyrosequencing (*i.e.*, after the PCR step that introduced the sample-specific tags). The amplicons were ligated into a plasmid vector, and 30 clones of each sample were sequenced. In each case, all sequences in the library were identical to the major phylotype obtained from the respective flagellate, which clearly identified the minor phylotypes in the corresponding pyrotag libraries as artifacts generated during the pyrosequencing process.

This interpretation is supported by the observation that the reads of Endomicrobia phylotype Tsph1, which was present in most *T. sphaerica* samples and overrepresented in the pyrosequencing run, were recovered with varying abundance from all other flagellate samples (Fig. 5; Supplementary Fig. S2). To test this quantitatively, we compared the number of reads for the major phylotype in each flagellate sample to the number of identical reads recovered from samples of other flagellate species (Fig. 6). The strong linear correlation corroborated the minor phylotypes in each sample not being real, but being artifacts caused by tag switching.

## Discussion

### Host specificity of Endomicrobia

In the present study, we showed that each of the *Trichonympha* cells from both *Z. nevadensis* and *R. santonensis* harbored a single phylotype of “*Ca.* Endomicobium trichonymphae”. The symbiont populations in each host lineage were phylogenetically distinct even if multiple host species were present in the same gut, corroborating the assumption that the apparent cospeciation between the partners in this symbiosis was caused by vertical (cytoplasmic) inheritance of the endosymbionts (23).

Although Ikeda-Ohtsubo and Brune (23) were able to document a congruent topology of the SSU rRNA trees of the symbiotic pairs, the relatively small number of sequences in their clone libraries (10–20 clones) did not allow us to exclude the presence of minor phylotypes of Endomicrobia within each *Trichonympha* cell. Moreover, since the libraries were obtained from flagellate suspensions containing numerous host cells (100–200 cells per sample), it remained possible that individual flagellates harbored a phylotype entirely different from that of the majority of the cells in the suspension.

Prompted by the finding that a large Endomicrobia clone library of *Z. nevadensis* (353 clones) contained a markedly larger number of 16S rRNA phylotypes than the *Trichonympha* species described for this flagellate (21), we reinvestigated the subject. Since the resolution of the 16S rRNA analysis was not sufficient due to the high similarity of the sequences, we used the more variable ITS region, harnessing the advantages of whole-genome amplification and next-generation sequencing technology to achieve a highly resolved analysis of their community structure based on the genomic DNA of the symbiont populations in individual host cells. Beyond the artifacts inherent to the pyrotag method (see below), we concluded that (i) each *Trichonympha* cell harbored a homogeneous population of Endomicrobia symbionts, (ii) *Trichonympha* cells of different species always harbored a phylogenetically distinct symbiont population, and (iii) the phylogenetic diversity of Endomicrobia in the two termites investigated in this study was caused by microdiversity in the *Trichonympha* populations that extended beyond the framework of the currently described species.

The present study could not completely exclude the possibility of an exchange of symbionts between individuals of the same (or closely related) *Trichonympha* populations present within the same gut. While the *Trichonympha* species in the wood-feeding cockroach *Cryptocercus punctulatus* still reproduce sexually during each molt of the host, this trait has been lost in the *Trichonympha* lineages of termites at an uncertain evolutionary time point (10). However, artificial feeding of *Z. angusticollis* with the molting hormone 20- hydroxyecdysone still appeared to trigger a sexual cycle in their *Trichonympha* flagellates (32), and such copulation events may still allow for the mixing of Endomicrobia phylotypes among host cells of the same species on rare occasions. Nevertheless, the strong and ongoing genome reduction demonstrated in “*Ca.* Endomicrobium trichonymphae” strain Rs-D17 (18) indicates the absence of genetic exchange and a frequent population bottleneck during transmission (50), a well-documented phenomenon in the intracellular symbionts in the bacteriocytes of aphids (33, 34).

### Endomicrobia populations in *Zootermopsis* and *Reticulitermes*

Assuming that all Endomicrobia species had the same copy number of rRNA genes, the Endomicrobia community in the gut of *Z. nevadensis* was dominated by phylotypes from *T. collaris* (43%) and *T. sphaerica* (31%). These outnumbered the diverse populations of “*Ca.* Endomicrobium trichonymphae” associated with *T. postcylindrica* and *T. campanula*, which accounted for another 19% of the community (Fig. 4B). Considering that the colonization density of Endomicrobia differed among the four *Trichonympha* phylotypes in *Z. nevadensis* (21), this is in reasonable agreement with the relative abundance of *Trichonympha* species in this termite, as reported by Kirby (25) who found that the three species (*T. postcylindrica* was not recognized as separate from *T. campanula*) were typically present in comparable numbers. The phylogenetic position of the remaining phylotypes (5% of the reads in the library) recovered only from the gut of *Z. nevadensis* and not from the selected flagellates (Fig. 4A) suggested that they represented Endomicrobia populations of *Trichonympha* lineages that were not included among the selected cells, but whose presence was indicated by the microdiversity of the ITS sequences obtained for each morphotype (Fig. 3).

**Fig. 1:**
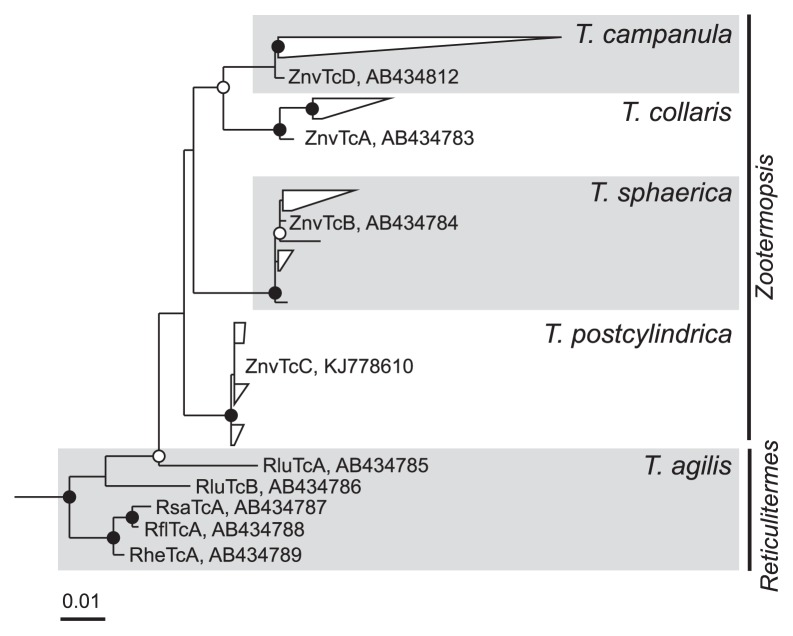
Diversity of 18S rRNA gene sequences of *Trichonympha* flagellates in *Zootermopsis nevadensis* (21), *Zootermopsis angusticollis* (grouped nodes) (45), and different *Reticulitermes* species (Rlu, *Reticulitermes lucifugus*; Rsa, *Reticulitermes santonensis*; Rfl, *Reticulitermes flavipes*; Rhe, *Reticulitermes hesperus*) (23). The maximum-likelihood tree was rooted with *Trichonympha* species of Clusters II and III. Circles indicate node support (○, >80%; ●, >95% bootstrap confidence).

In the case of the *T. agilis* cells selected from the gut of *R. santonensis*, the microdiversity of Endomicrobia (Fig. 4A) reflected that of the host flagellates (Fig. 3), which indicated the strict host specificity of the endosymbionts. Kirby (25) reported that, with the exception of *Reticulitermes lucifugus*, all *Reticulitermes* species investigated harbored only a single species of *Trichonympha*, which was in agreement with the number of SSU rRNA genes of *Trichonympha* species obtained from different *Reticulitermes* species (Fig. 1). However, the ITS sequences of the selected flagellates revealed the presence of two closely related phylotypes in *R. santonensis* (Fig. 3), which is consistent with the two phylotypes of “*Ca.* Endomicrobium trichonymphae” obtained from these samples (Fig. 4).

The reads obtained for the amplified ITS regions of the individual flagellates of *T. campanula* often contained traces of sequence polymorphism, suggesting that the rRNA gene clusters had multiple and slightly divergent copies. To date, there has been no genome information for any termite gut flagellate; however, the draft genome of the distantly related parabasalid *Trichomonas vaginalis* contains 254 copies of the 18S rRNA gene (8). ITS regions experience low selective constraint and are considered to evolve rapidly by large insertions and/or deletions (14, 41).

### Methodological pitfalls of pyrotag sequencing

In amplicon pyrosequencing, the addition of sequence tags to the amplified target sequences allows for the parallel processing of numerous samples in the same sequencing run (17). The tags are typically added to both ends of the amplicons because this allows for the identification of cases of tag switching (2), which result in impossible tag combinations at each terminus and may affect a large proportion of the reads in pyrotag libraries (7, 49).

However, tag switching can be recognized and removed during quality control only if both termini of the amplicon are present in the same read. If the length of the amplicon exceeds the read length, a common problem with most sequencing platforms, it is not possible to identify mistagged sequences without any prior information. In this case, they either go entirely unnoticed or are purged from the dataset by omitting all reads that do not pass a certain frequency threshold (*e.g.*, 46).

**Fig. 2:**
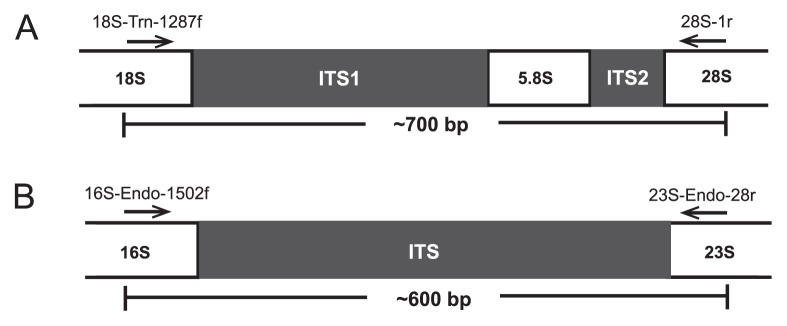
Strategy used for PCR amplification of ITS regions of (A) *Trichonympha* flagellates and (B) their Endomicrobia symbionts. The primers, their target positions in the proximal regions of the flanking rRNA genes, and the resulting amplicon length are indicated.

**Fig. 3:**
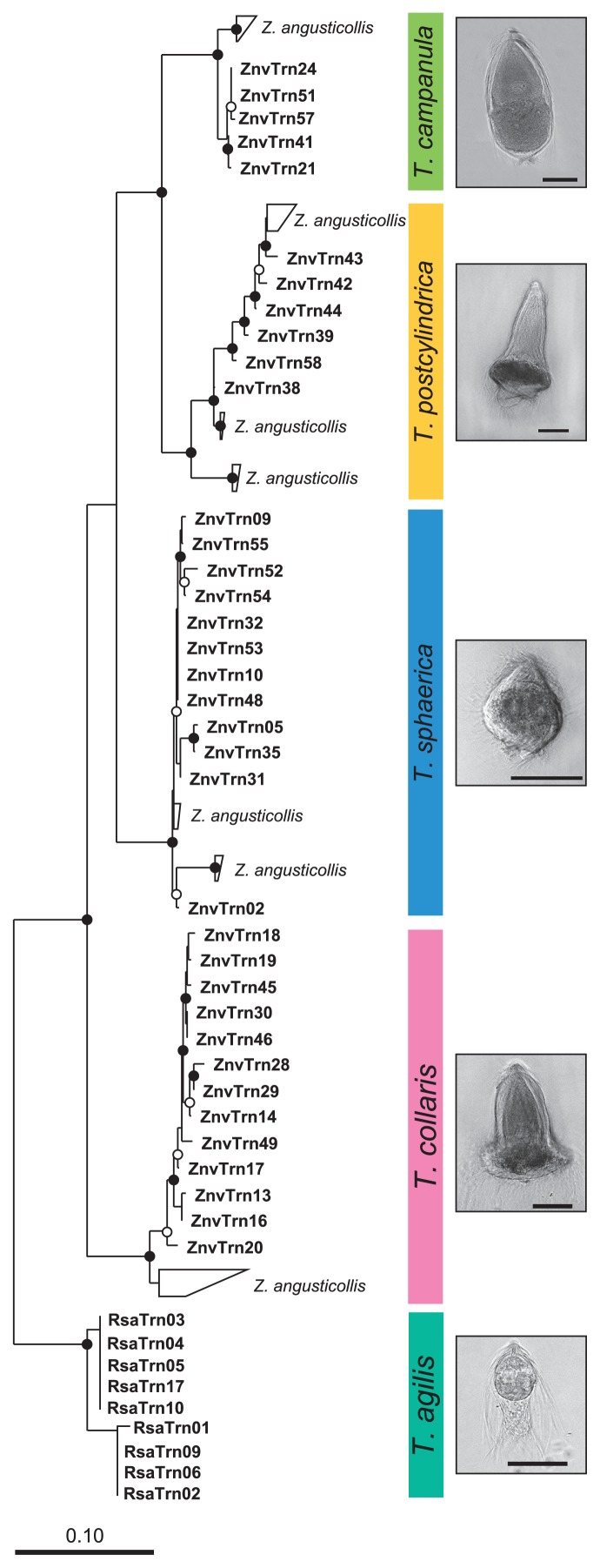
Neighbor-joining tree of the ITS sequences obtained from selected flagellates, illustrating the phylogenetic diversity of the *Trichonympha* species from *Zootermopsis nevadensis* (this study) and their relationship to those from *Zootermopsis angusticollis* (45; sequences are grouped). The tree is based on an alignment of 450 base positions and was rooted with the ITS sequences of *Trichonympha agilis* from *Reticulitermes santonensis*. Phase-contrast photomicrographs illustrate the morphology of the respective species (scale bars=50 μm). Circles indicate node support (○, >80%; ●, >95% bootstrap confidence).

**Fig. 4:**
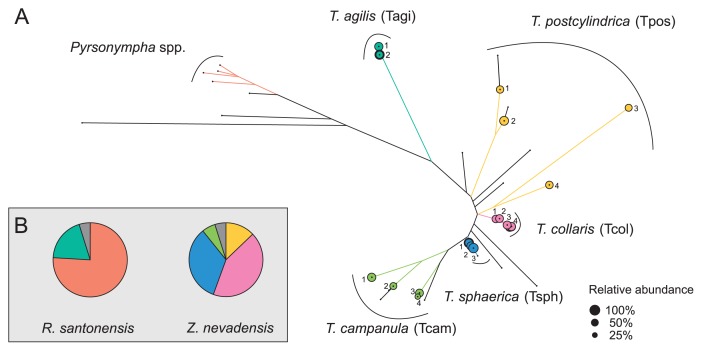
(A) Maximum-likelihood tree of all Endomicrobia phylotypes (99% sequence similarity) detected in whole-genome amplification (MDA) products of all single flagellate cells. Branches shared by phylotypes from the same host species are color coded; the area of the circles indicates the relative abundance of the major phylotype in each library. Black dots indicate the position of phylotypes that originated from whole-gut samples of *Zootermopsis nevadensis* or *Reticulitermes santonensis*. (B) Pie charts indicate the relative abundance of the different Endomicrobia clusters in the whole gut samples. The colors represent the major phylotypes from selected *Trichonympha* cells; phylotypes from whole-gut samples are shown in grey. Phylotypes in the *Pyrsonympha* cluster were identified based on the sequences retrieved from selected flagellates from *R. santonensis* (unpublished results).

**Fig. 5:**
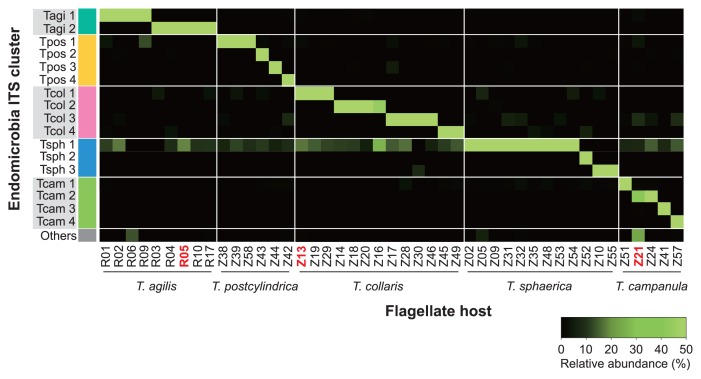
Heatmap of the relative abundance of different phylotypes of Endomicrobia within pyrotag libraries of all single flagellate cells. The samples were ordered according to the clusters indicated in Fig. 2. The samples that were checked for purity by cloning and Sanger sequencing are in red.

**Fig. 6:**
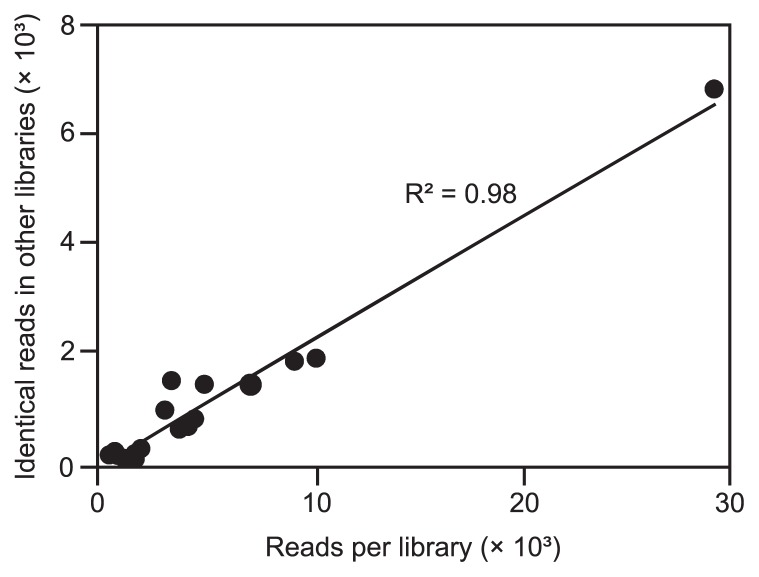
Correlation of the number of reads for the major phylotype in each pyrotag library of individual flagellates to the number of identical reads recovered from libraries of other flagellate species. Each dot represents the results obtained for an individual sample.

In the present study, the necessities of primer design dictated an amplicon length of approximately 600 bp (the ITS region of Endomicrobia; Fig. 2B), which exceeded even the read length of the 454 Titanium technology (up to 400 bp) (15). However, the low diversity of the Endomicrobia community and the highly improbable presence of identical phylotypes in host flagellates of different termites allowed us to identify reads with suspicious tags. Their frequency amounted to 15% of the reads in the entire sequencing run (Supplementary Fig. S2), which is at the upper end of the proportion of mistagged reads reported in previous studies (49). The complete absence of these reads from the processed samples prior to the sequencing run excludes them resulting from contaminated tags and indicated that they originated during the pyrosequencing step, possibly from tag switching during emulsion PCR, as suggested previously (7). Since the number of mistagged reads in each sample depends on the number of reads carrying a particular tag (Fig. 6; Supplementary Fig. S2), mistagged reads cannot be removed simply by applying a frequency threshold. In cases that are highly sensitive to tag switching, *e.g.*, the identification of core communities shared across multiple samples, such artifacts have to be excluded using an appropriate experimental design. Otherwise, the simultaneous presence of OTUs in different samples of a pyrotag run has to be interpreted with the necessary caution (12).

## Supplementary Information
