# Supplementary material for: Population Structure of Endomicrobia in Single Host Cells of Termite Gut Flagellates (Trichonympha spp.)
Source: Microbes Environ. 2015 Feb 26;30(1):92–8. doi: 10.1264/jsme2.ME14169 (PMC4356469; doi:10.1264/jsme2.ME14169)
Supplement: Supplementary file 1 [file 30_92_s1.pdf]

SUPPLEMENTARY INFORMATION

Population structure of Endomicrobia in single host cells of termite gut flagellates (*Trichonympha* spp.)

HAO ZHENG, CARSTEN DIETRICH, CLAIRE L. THOMPSON, KATJA MEUSER, ANDREAS BRUNE

Department of Biogeochemistry, Max Planck Institute for Terrestrial Microbiology, Karl-von-Frisch-Str. 10, 35043 Marburg, Germany

Fig. S1. Sequence polymorphism in the ITS region of *Trichonympha campanula*, illustrated by a trace file of the direct Sanger sequencing of the PCR product obtained from a single-flagellate MDA sample (ZnvTrn21). The positions of multiple peaks (indicated by bars) are affected by the sequencing direction but always start in the ITS region, suggesting the presence of both substitutions and indels. For phylogenetic analysis, we used the consensus of the sequences obtained forward and reverse primers, always selecting the stronger signal at the polymorphic positions.

Fig. S2. Composition of Endomicrobia ITS libraries obtained by pyrotag analysis of the whole-genome amplification products of individual *Trichonympha* cells. The color code indicates the host species of the major phylotypes. Other phylotypes are shown in grey. The area of each circle represents the number of reads in each library. The names of the samples that were checked for purity by cloning and Sanger sequencing are in red.

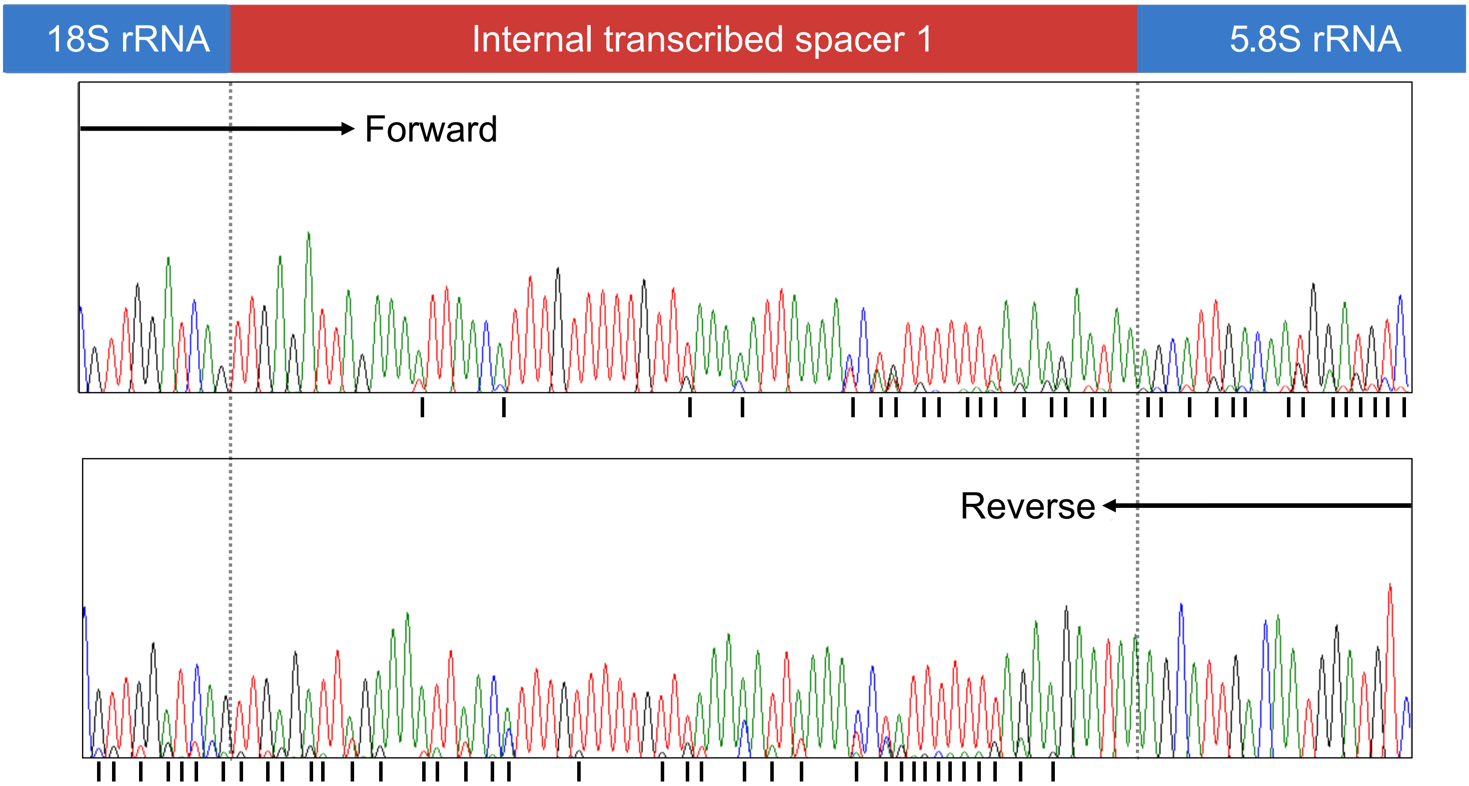

Fig. S1 Hao Zheng *et al.*

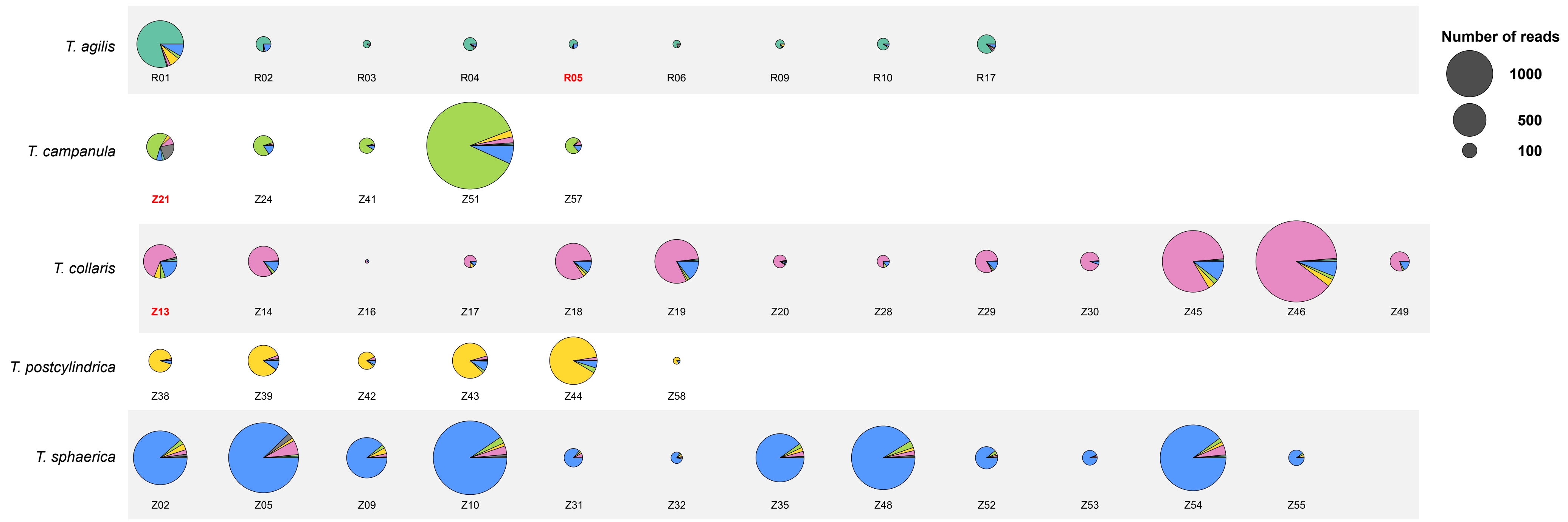

Fig. S2 Hao Zheng *et al.*
